# Involvement of PRRSV NSP3 and NSP5 in the autophagy process

**DOI:** 10.1186/s12985-019-1116-x

**Published:** 2019-01-28

**Authors:** Wei Zhang, Keren Chen, Yang Guo, Yaosheng Chen, Xiaohong Liu

**Affiliations:** State Key Laboratory of Biocontrol, School of Life Sciences, Sun Yat-sen University, Guangzhou, 510006 People’s Republic of China

**Keywords:** Porcine reproductive and respiratory syndrome virus, Autophagosomes, Endoplasmic reticulum, NSP3 and NSP5, Cytoplasmic domain

## Abstract

**Background:**

Autophagy is an essential process in eukaryotic cells in which autophagosomes form to deliver cellular organelles and long-lived proteins to lysosomes for degradation. Many studies have recently identified the regulatory mechanisms involved in the interaction between viral infection and autophagy.

**Methods:**

LC3 turnover and the proteins in the endoplasmic reticulum (ER) stress pathway were investigated using western blot analysis. The formation and degradation of autophagosomes were detected using immunofluorescence staining.

**Results:**

Autophagy was activated by porcine reproductive and respiratory syndrome virus (PRRSV) NSP3, NSP5 and NSP9, which are two transmembrane proteins and an RNA-dependent RNA polymerase, respectively. The formation of autophagosomes was induced by NSP3 and NSP5 and developed from the ER; the fusion of these autophagosomes with lysosomes was limited. Although NSP3 and NSP5 are ER transmembrane proteins, these proteins did not activate the ER stress signaling pathways. In addition, the cytoplasmic domain of NSP3 plays a pivotal role in activating autophagy.

**Conclusions:**

The data presented in this study reveal an important relationship between PRRSV NSPs and autophagy and provide new insights that improve our understanding of the involvement of PRRSV NSPs in the autophagy process.

**Electronic supplementary material:**

The online version of this article (10.1186/s12985-019-1116-x) contains supplementary material, which is available to authorized users.

## Background

Porcine reproductive and respiratory syndrome virus 2 (PRRSV-2) is a member of the genus *Arterivirus*, which includes equine arteritis virus (EAV), lactate dehydrogenase-elevating virus (LDV), and simian hemorrhagic fever virus (SHFV) [1]. PRRSV infection leads to severe morbidity and mortality worldwide; the main clinical manifestations are reproductive disorders, such as miscarriage, stillbirth, and the delivery of mummified fetuses in pregnant sows, as well as respiratory symptoms in pigs of various ages (particularly piglets) [2, 3]. PRRSV is an enveloped, single-stranded positive-strand RNA virus with a genome of approximately 15.4 kb that includes at least 9 open reading frames (ORFs); ORF1a and ORF1b encode nonstructural proteins (NSPs), and ORF2-ORF7 encode structural proteins [4, 5]. These NSPs are involved in viral replication and play key roles in regulating genomic transcription [6]. Among these NSPs, NSP3 and NSP5 are predicted to be transmembrane proteins and NSP9 possesses viral RNA-dependent RNA polymerase activity [7]. PRRSV is usually grown in Marc-145 cells, which originated from monkey kidney epithelial cells.

Autophagy occurs in eukaryotic cells and is a process by which cell homeostasis is maintained through the degradation of proteins and organelles [8, 9]. Generally, autophagy is triggered in cells stimulated with external factors, such as starvation or viral infection; the marker of autophagy is the formation of autophagosomes [10]. The integrated autophagy course primarily comprises three processes: first, the formation of autophagosomes; then, the fusion of autophagosomes with lysosomes; and last, degradation of the cargo in lysosomes. The first step of the autophagy process is that a membrane possibly derived from the endoplasmic reticulum (ER) elongates, becomes curled, and then surrounds part of the cytoplasm, followed by the degradation of organelles, proteins and other cellular components. In this process, two proteins, namely, ATG12 and ATG5, form complexes via ubiquitin-like conjugation [11, 12]. Furthermore, ATG16 interacts with the ATG12-ATG5 complex to form the ATG12-ATG5-ATG16 protein complex, which plays a significant role in forming autophagosomes [13]. Finally, lysosomes fuse with mature autophagosomes to form autophagolysosomes; the contents of the autophagosome are degraded, and the substances inside the autophagolysosomes are delivered to the cytoplasm for transformation into amino acids and energy for cytoplasmic metabolism [14].

Generally, autophagy is accompanied by ER stress, which is induced by the accumulation of unfolded or misfolded proteins in the ER [15]. The ER is the main site of protein synthesis, lipid production and calcium ion storage in eukaryotic cells. A variety of proteins in the ER must be folded, assembled, processed, packaged and transported to the Golgi apparatus [16]. Once the cells are stimulated with internal and external factors, the balance between ER morphology and function is affected by the changes in molecular biochemistry. Protein processing and transport are blocked, and a large number of unfolded or misfolded proteins accumulate in the ER, leading to the unfolded protein response (UPR) [17–19], which operates through three signaling pathways that play significant roles in regulating autophagy during viral infection [20, 21]. The ER stress response was recently shown to be induced by PRRSV infection, leading to the activation of JNK signaling pathways [22].

In the past decade, a number of studies have reported a connection between autophagy and viral proteins [23, 24]. The HCV polymerase NS5B was recently reported to colocalize with NS4B and interact with ATG5 to initiate autophagosome formation [25]. In addition, HBx promotes autophagy and prevents the fusion of autophagosomes with lysosomes to maintain the accumulation of solutions in the autophagosomes [26, 27]. Recent studies examining the relation between autophagy and PRRSV revealed that PRRSV induced incomplete autophagy and that autophagy enhances the replication of PRRSV [28–30]. Additionally, PRRSV infection promotes mitochondrial fission and activates apoptosis [31–33]; however, few studies have examined the relations between PRRSV NSPs and autophagy to date.

In this study, we detected the involvement of PRRSV NSPs in autophagy in transfected cells for the first time. PRRSV NSP3, NSP5 and NSP9 induce the formation of autophagosomes. Additionally, NSP3 and NSP5 are ER transmembrane proteins, but these two NSPs trigger limited ER stress, and the autophagosomes induced by these two NSPs are derived from the ER and accumulate autophagic cargo to prevent fusion with lysosomes. Furthermore, an NSP3 truncation mutant consisting of only the hydrophobic domains did not induce the formation of autophagosomes. Our findings revealed the important functions of PRRSV NSPs in autophagy.

## Materials and methods

### Cells and virus

Cells of the African green monkey kidney epithelial cell line Marc-145 purchased from ATCC were cultivated in Dulbecco’s Modified Eagle’s Medium (DMEM, Invitrogen) supplemented with 10% fetal bovine serum (FCS) at 37 °C in a 5% CO_2_ atmosphere. HBSS (Hank’s Balanced Salt Solution) was purchased from Sigma and can induce autophagy. The PRRSV strain CH-1a (the first PRRSV2 strain isolated from China) was provided by Dr. Guihong Zhang at the South China Agricultural University, China.

### Antibodies

Cellular DNA was stained with 4′,6′-diamidino-2-phenylindole dihydrochloride (DAPI), and mitochondria were stained with MitoTracker Green. Rabbit anti-LC3, anti-calnexin, and anti-GAPDH; mouse anti-ATG5; Alexa Fluor 488-conjugated anti-rabbit IgG; Alexa Fluor 488-conjugated anti-mouse IgG; and Alexa Fluor 647-conjugated anti-mouse IgG secondary antibodies were purchased from Cell Signaling Technology (Beverly, MA). A mouse anti-mCherry antibody was obtained from Abcam (ab167453). A PRRSV nucleocapsid (N) antibody was purchased from Jeno Biotech Inc. (Chuncheon). The mouse monoclonal anti-dsRNA antibody was purchased from Scicons (Hungary).

### Plasmid construction and DNA transfection

Viral RNA was isolated with TRIzol (Invitrogen), and cDNAs were obtained using a Reverse Transcription Kit (Promega). PCR primers were designed to amplify the PRRSV Nsp transcripts (Additional file 1: Table S1) using the PRRSV CH-1a cDNA. The PCR fragments were cloned into a pmCherryN1 (Clontech, 632,523) expression vector. The truncated PRRSV Nsp3 mutant was constructed using a PCR primer pair (Additional file 1: Table S1). All multiple cloning digestion sites were selected for digestion with XhoI and BamHI. Marc-145 cells were transfected with 1 μg of the constructed plasmids using Lipofectamine 3000 (Invitrogen).

### Immunofluorescence staining

Marc-145 cells were cultured on glass coverslips, followed by transfection with the individual plasmids for 24 h. After three washes with phosphate-buffered saline (PBS), cells were fixed with 4% paraformaldehyde (PFA) for 15 min at room temperature. After three washes with PBS, the cells were permeabilized with 0.5% Triton X-100 for 5 min and blocked with 1% bovine serum albumin (BSA) in PBS for one hour at room temperature. Next, the coverslips were incubated with primary antibodies (diluted 1:500) in PBS containing 1% BSA for 2 h, followed by an incubation with an Alexa Fluor-conjugated secondary antibody (diluted 1:1000) for 1 h at room temperature in the dark. After three washes with PBS, cellular nuclei were stained with DAPI in the dark for 5 min at room temperature. Next, the coverslips were washed with PBS, mounted on the microscope slides with antifade mounting medium, and observed under a Leica TCS SP5 confocal microscope.

### Immunoblotting

Cells were washed with PBS, and total protein was obtained using SDS cell lysis buffer (1% SDS and 10 mM Tris, pH 6.8) containing 1 mM phenylmethylsulfonyl fluoride (PMSF) and heating for 5 min. A volume of lysate containing 20 μg of protein was separated on 12% acrylamide gels in a Bio-Rad system. Then, proteins were transferred to a polyvinyl difluoride (PVDF) membrane and blocked with 3% BSA in Tris-buffered saline including Tween 20 (TBST) for 50 min. After blocking, the PVDF membrane was incubated with the primary antibody for 2 h, followed by incubation with the HRP-conjugated secondary antibodies. Images were captured using an Image Station 4000 mm PRO System and Image Station 4000 mm PRO software.

### Xbp1 PCR

Cells were collected, and total RNA was extracted using TRIzol (Invitrogen). Two micrograms of RNA was obtained with the Reverse Transcription Kit as described above. The synthesized cDNAs were amplified with specific primers (Additional file 1: Table S1). The final products were visualized on a 1.5% agarose gel that was electrophoresed at 110 V for 45 min.

### Statistical analysis

The Pearson correlation coefficient per cell was quantified using Image-Pro Plus software. Data are presented as means ± standard errors. Statistical significance was determined by performing Student’s t-tests. *P* values < 0.05 were considered statistically significant.

## Results

### The induction of autophagosome formation following PRRSV infection

**Fig. 1:**
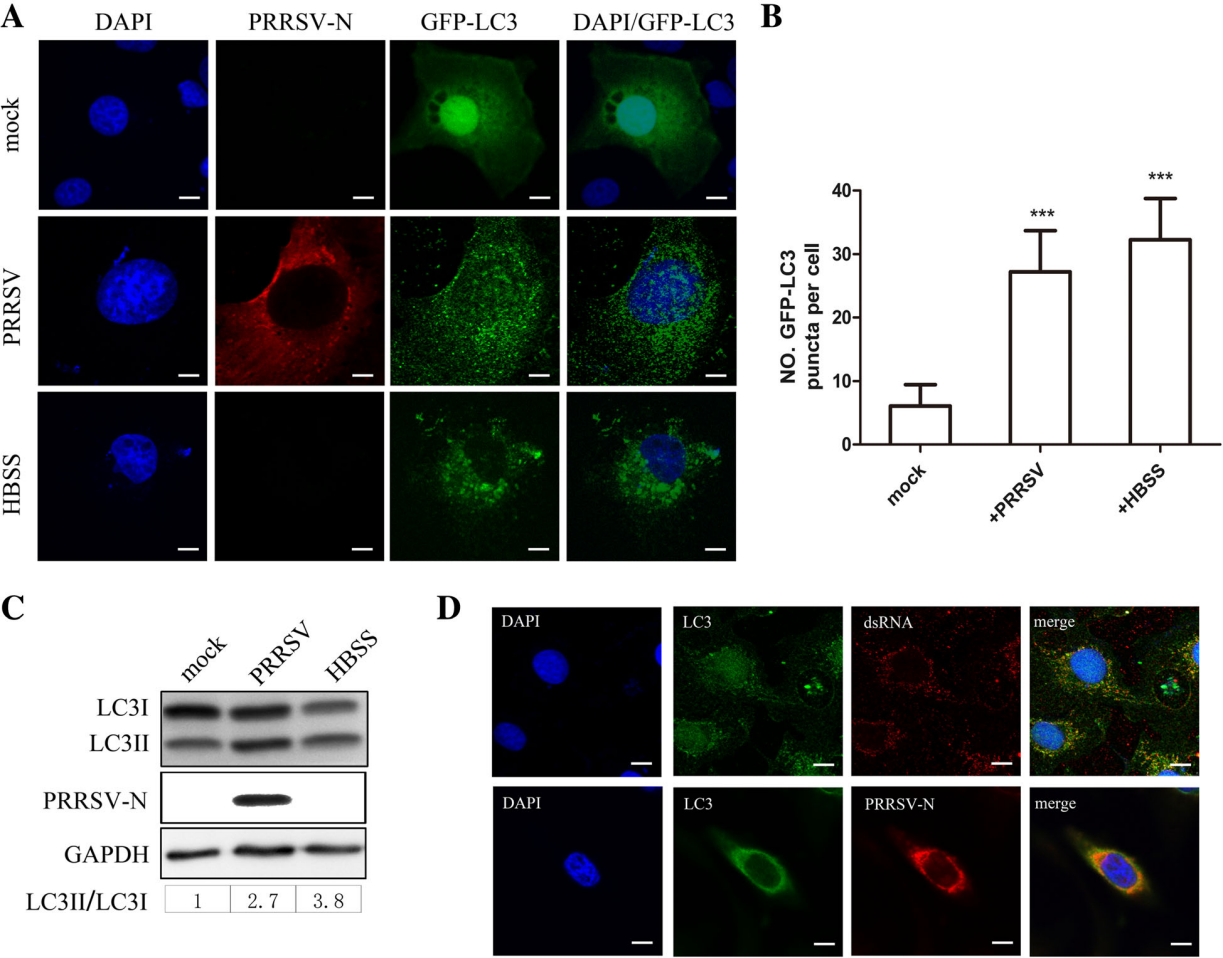
The distribution of autophagy proteins in PRRSV-infected Marc-145 cells. **a** Marc-145 cells were transfected with GFP-LC3 plasmids and cultured with either DMEM or HBSS media for 4 h or were infected with PRRSV CH-1a for 24 h. Fixed cells were observed under a fluorescence microscope. Nuclei were stained with DAPI (blue), and virions were stained with an antibody against the PRRSV-N protein (red). Scale bars: 10 μm. **b** Statistical analysis of the number of GFP-LC3 puncta in mock, HBSS-treated or PRRSV-infected cells; the number represents GFP-LC3 puncta per cell; data are presented as means ± SD, *n* = 30. **c** LC3 conversion in Marc-145 cells. Marc-145 cells were mock infected, infected with PRRSV for 24 h or cultured in HBSS media. Cells lysates were subjected to immunoblotting. The ratio of LC3II/LC3I reflects the level of autophagy. **d** Marc-145 cells were infected with PRRSV for 24 h, and fixed cells were observed under a fluorescence microscope. Nuclei were stained with DAPI (blue); dsRNA and PRRSV-N are labeled in red, and endogenous LC3 is labeled in green. Scale bars: 10 μm

The GFP-LC3 plasmid, which expressed the LC3 protein tagged at its N terminus with the fluorescent protein GFP, was used to monitor the formation of autophagosomes by indirect immunofluorescence [14]. GFP-LC3 was transfected into cells for 24 h, and transfection efficiency was 50–70%. Cells were then infected with PRRSV CH-1a. At 24 h.p.i., the infected cells were fixed, and GFP-LC3 puncta were observed to assess the formation of autophagosomes. As shown in Fig. 1a and b, compared to the accumulation of GFP-LC3 puncta in the cytoplasm of mock-infected cells, the accumulation of these puncta in the cytoplasm of HBSS-treated and PRRSV-infected cells suggested that PRRSV induced the formation of autophagosomes. LC3 conversion is a hallmark of autophagy; therefore, the conversion of LC3 was assessed by immunoblotting and the levels of LC3II/LC3I were examined to assess the induction of autophagy. Marc-145 cells were infected with PRRSV CH-1a at 24 h.p.i. or were cultured with HBSS for 4 h as a positive control. As shown in Fig. 1c, compared to the LC3II/LC3I ratio in the mock-infected cells, the ratio was increased in the infected Marc-145 cells. We explored whether PRRSV dsRNA and N proteins were associated with autophagosomes using confocal microscopy to identify whether the autophagosomes induced by PRRSV were related to viral replication or assembly. As depicted in Fig. 1d, the majority of the LC3 protein was colocalized with dsRNA and N proteins, indicating that these autophagosomes provide the site for PRRSV replication and assembly.

### PRRSV NSP3 and NSP5 induce autophagosome formation

**Fig. 2:**
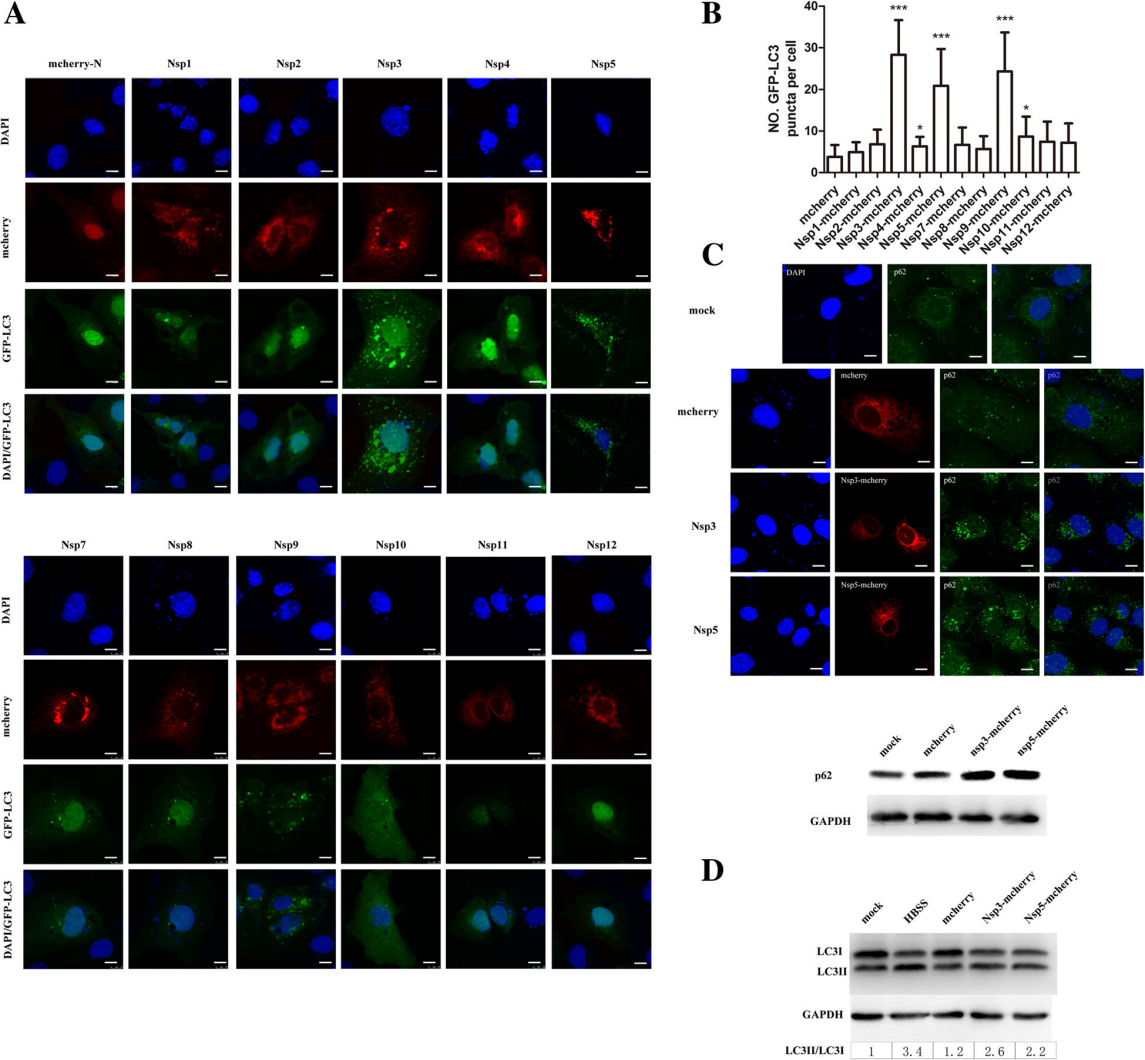
The formation of autophagosomes induced by PRRSV NSP3 and NSP5. **a** Marc-145 cells were transfected with GFP-LC3 and plasmids encoding the individual PRRSV Nsps; each Nsp was cloned into an mCherry-N plasmid, and cells transfected with mCherry-N were used as a negative control. Twenty-four hours after transfection, cells were harvested and visualized under a fluorescence microscope. Nuclei were stained with DAPI, and the number of GFP-LC3 puncta, which is related to autophagy, was analyzed using GraphPad software. Scale bars: 10 μm. **b** Statistical analysis of the number of GFP-LC3 puncta in cells expressing mCherry-N and each Nsp; the numbers represent GFP-LC3 puncta per cell; data are presented as means ± SD, n = 30. **c** Cells were transfected with mCherry Nsp3-mCherry and Nsp5-mCherry for 24 h; the endogenous p62 protein was stained with a green fluorophore. Cell lysates were subjected to immunoblotting. Scale bars: 10 μm. **d** LC3 protein conversion. Marc-145 cells were mock infected; treated with HBSS for 4 h or transfected with mCherry, Nsp3-mCherry, or Nsp5-mCherry; and then harvested and lysed. Extracts were analyzed by western blotting using an anti-LC3 antibody. The ratio of LC3II/LC3I reflects the level of autophagy

PRRSV non-structural proteins play an important role in virus replication and assembly and use the substances in the cells to influence cell life activities. Because PRRSV induced the formation of autophagosomes, we further explored which PRRSV NSPs played important roles in this process. Eukaryotic expression vectors carrying the Nsp cDNAs with an N-terminal mCherry tag were constructed and transfected into Marc-145 cells (Additional file 2: Figure S1). As shown in Fig. 2a and b, the GFP-LC3 puncta accumulated in Nsp3-mCherry-, Nsp5-mCherry- and Nsp9-mCherry-transfected cells. NSP9 is an RNA-dependent RNA polymerase (RdRp) that plays important roles in viral transcription and replication, and NSP3 and NSP5 are predicted to be transmembrane proteins; these proteins are anchored on the cytoplasmic membrane and are part of the membrane-bound replication and transcription complex. Furthermore, LC3 levels were detected using immunoblotting to determine the effects of the two transmembrane proteins on autophagy. p62/sequestosome-1 is a protein that can bind to LC3 as a scaffold protein or a signaling adapter and may be increased at the beginning of autophagy process and degraded gradually. Based on the data presented in Fig. 2c, immunoblotting and immunofluorescence assay showed that the expression of the p62 protein was increased, indicating that NSP3 and NSP5 of PRRSV induced the formation of autophagosomes. As shown in Fig. 2d, a higher LC3II/LC3I ratio was observed in Nsp3-mCherry- and Nsp5-mCherry-transfected cells than in mock-infected cells. We identified some of the effects of these two transmembrane proteins on cell autophagy to determine whether the NSPs affected cytoplasmic membrane fusion.

### The autophagosomes induced by NSP3 and NSP5 are derived from the ER

**Fig. 3:**
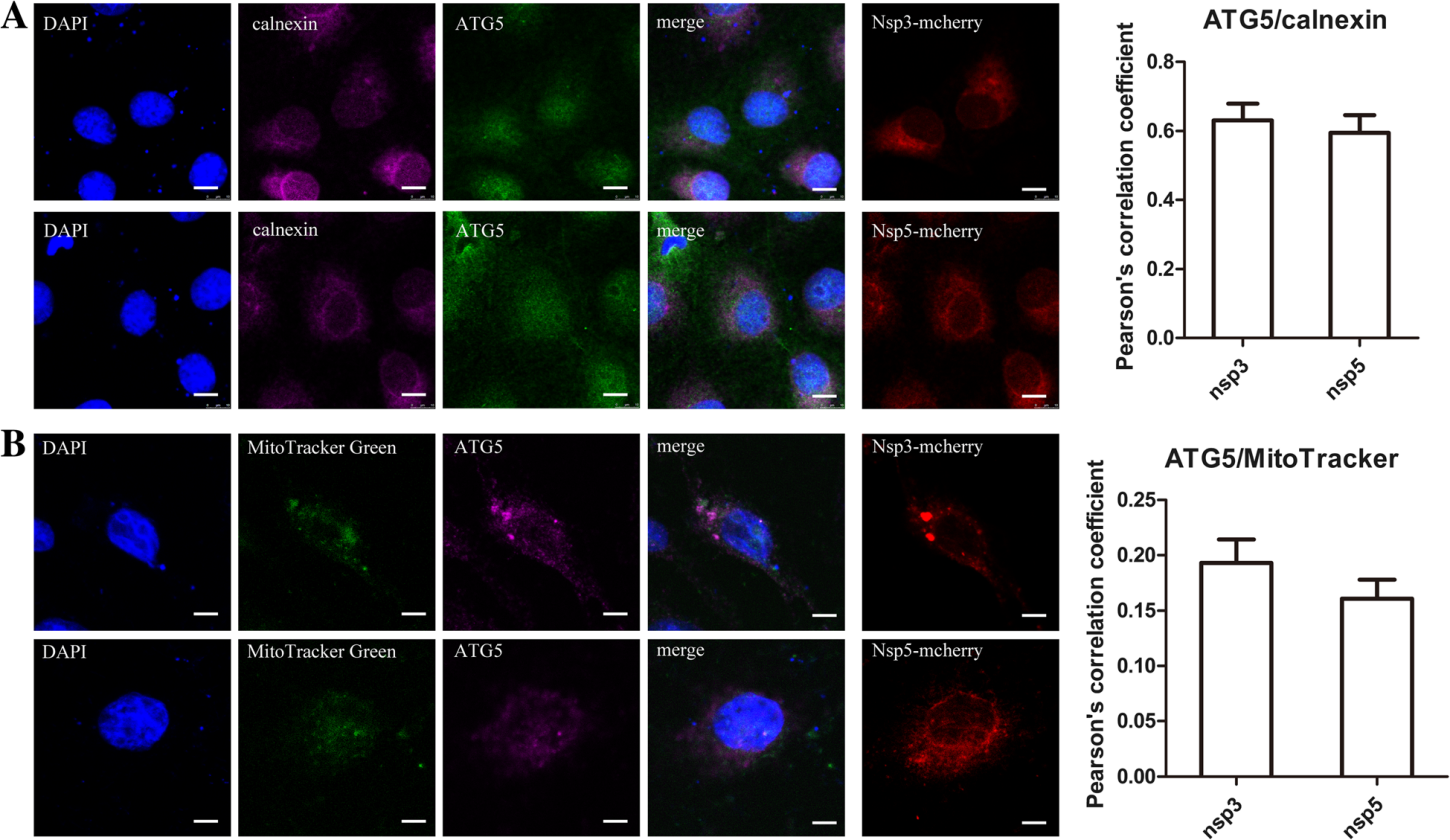
Autophagosomes originated from the ER but not mitochondria. **a** Marc-145 cells were transfected with Nsp3-mCherry and Nsp5-mCherry for 24 h; ATG5 is labeled in green, and nuclei were stained with DAPI (blue). Scale bars: 10 μm. Statistical analysis of the Pearson correlation coefficient for calnexin and ATG5. The Pearson colocalization coefficient is presented as the ratio of punctate signals of calnexin that were positive for ATG5. **b** Nsp3-mCherry and Nsp5-mCherry were expressed in Marc-145 cells. Twenty-four hours after transfection, cells were cultured with MitoTracker Green, a marker of mitochondria, and were then fixed and immunostained with an anti-ATG5 antibody (pink). Nuclei were stained with DAPI (blue). Scale bars: 10 μm. Statistical analysis of the Pearson correlation coefficient for MitoTracker Green and ATG5. The colocalization coefficient is presented as the ratio of fluorescent signals of MitoTracker Green that were negative for ATG5

Generally, in the early stage of autophagy, ATG5 complexes with ATG12 and ATG16L1 to form the ATG12-ATG5-ATG16L1 complex [34], and this complex attaches to nascent phagophores, which are the precursors of mature autophagosomes that are involved in regulating membrane morphology changes [35]. Therefore, endogenous ATG5 was confirmed at the site of immature autophagosomes. Autophagosome membranes have been shown to originate from the mitochondria [36] or ER [37]. Cells transfected with Nsp3-mCherry and Nsp5-mCherry were fixed and stained for ATG5 and calnexin, an ER marker. The value of overlap of the two different channels was calculated using Pearson’s correlation coefficient, and a value exceeding 0.5 was determined to indicate colocalization. As shown in Fig. 3a, ATG5 puncta were arranged in reticular structures and colocalized with calnexin in the cells expressing NSP3 and NSP5. Combined with the subsequent findings that PRRSV NSP3 and NSP5 were localized on the ER, these results suggested that the ATG5 puncta were derived from the ER. Little colocalization of the fluorescently labeled ATG5 proteins with MitoTracker Green, a mitochondrial marker, was observed in cells transfected with Nsp3-mCherry and Nsp5-mCherry (Fig. 3b). Therefore, the autophagosomes induced by PRRSV NSP3 and NSP5 originate from the ER but not from the mitochondria.

### Autophagosomes induced by PRRSV NSP3 and NSP5 did not fuse with lysosomes

**Fig. 4:**
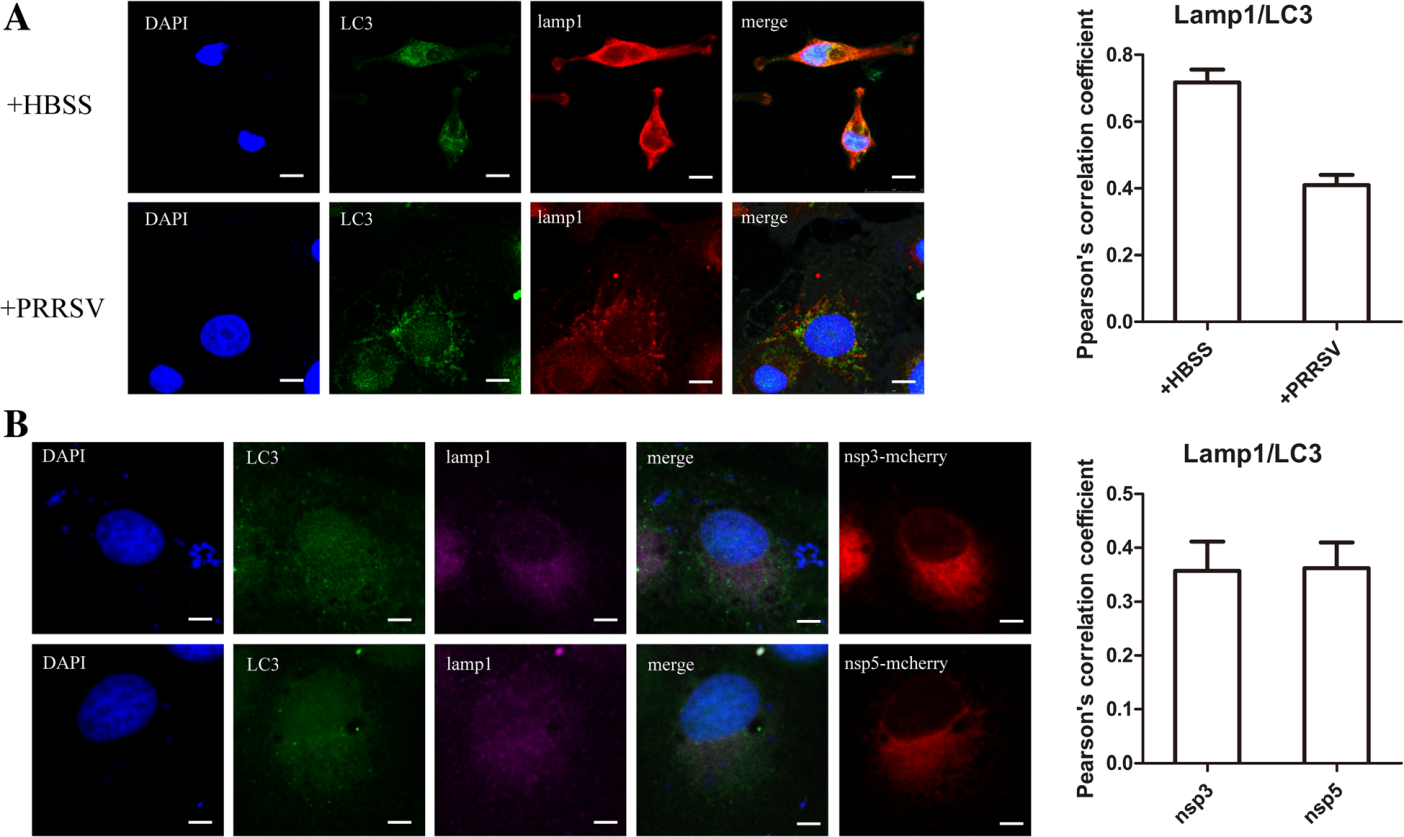
Lysosome fusion with autophagosomes induced by NSP3 and NSP5. **a** Marc-145 cells were starved for 4 h or infected with PRRSV CH-1a for 24 h, and the endogenous LC3 and LAMP1 proteins were labeled with green and red fluorophores, respectively. Scale bars: 10 μm. Statistical analysis of the Pearson correlation coefficient for LC3 and LAMP1. The colocalization coefficient is presented as the ratio of fluorescent signals of LC3 that were positive for LAMP1. **b** Nsp3-mCherry or Nsp5-mCherry was transfected into cells for 24 h. Cells were fixed, stained with fluorescent dye-conjugated LC3 and LAMP1 antibodies, and observed under a fluorescence microscope. Scale bars: 10 μm. Statistical analysis of the Pearson correlation coefficient for LC3 and LAMP1. The colocalization coefficient is presented as the ratio of fluorescent signals of LC3 that were negative for LAMP1

After mature autophagosomes are formed, they fuse with lysosomes and form autolysosomes to degrade the insoluble substances inside the autophagosomes. We labeled Nsp3- and Nsp5-transfected cells with lysosome-associated membrane protein 1 (LAMP1), a lysosomal marker, and the mature autophagosome marker LC3 to investigate the fate of autophagosomes and to obtain a better understanding of the role of these autophagosomes induced by the two NSPs. As shown in Fig. 4a, an overlap of LC3 and LAMP1 was observed in serum-starved Marc-145 cells. As shown in Fig. 4b, in nsp3 or nsp5 transfected cells, LAMP1 failed to colocalize with LC3, and the Pearson correlation coefficient was much lower than that in serum-starved cells, suggesting that lysosomes did not fuse with the autophagosomes induced by the two Nsps, as with PRRSV-infected cells. These results suggest that the autophagosomes induced by NSP3 and NSP5 are derived from the ER, but autophagosome-lysosome fusion was limited.

### NSP3 and NSP5 do not induce ER stress

**Fig. 5:**
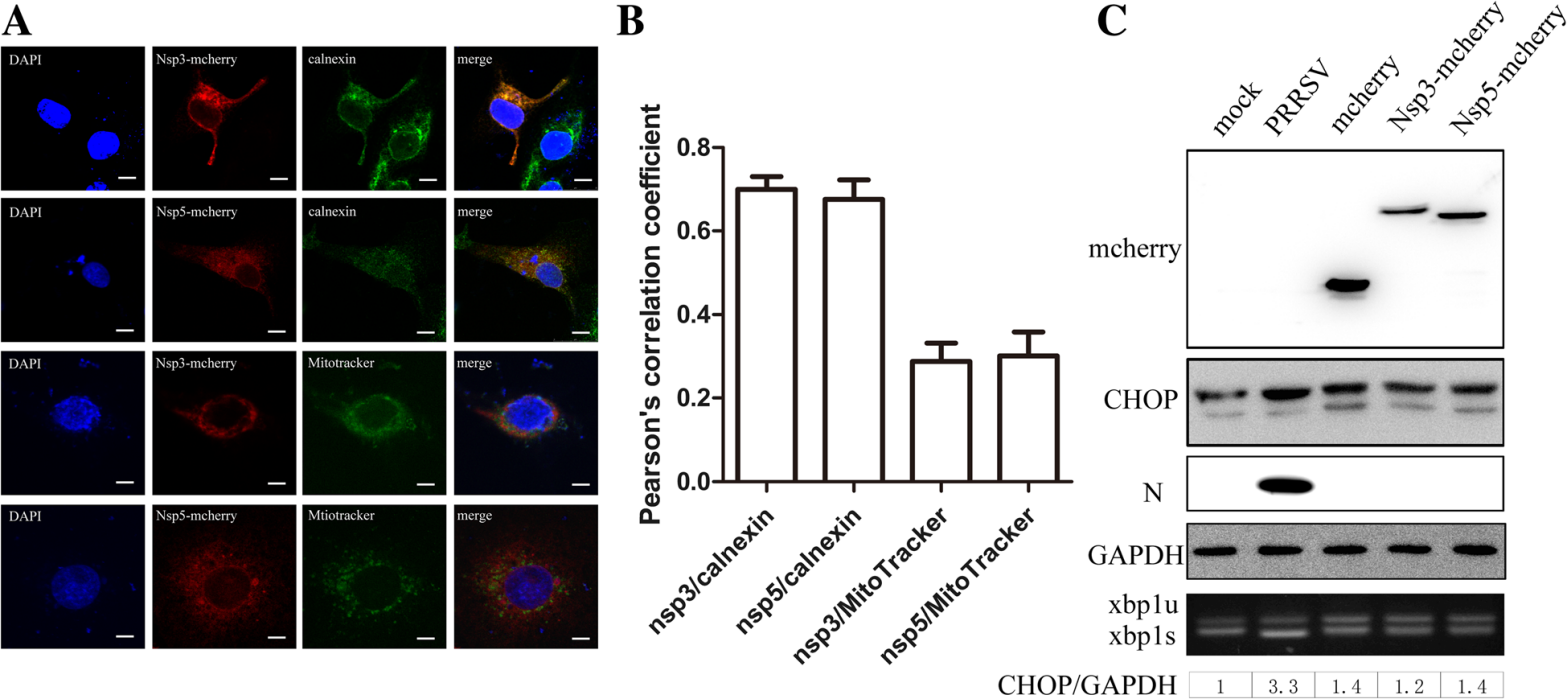
**a** NSP3 and NSP5 were located in the ER but not the mitochondria. Nsp3-mCherry and Nsp5-mCherry were expressed in Marc-145 cells; the ER was stained with an antibody against endogenous calnexin (green), and mitochondria were stained with MitoTracker Green. Scale bars: 10 μm. **b** Statistical analysis of the Pearson correlation coefficients for calnexin/MitoTracker and NSP3/NSP5. The colocalization coefficient is presented as the ratio of fluorescent signals of calnexin that were positive for NSP3/NSP5. The colocalization coefficient is the ratio of fluorescent signals of calnexin that were negative for NSP3/NSP5. **c** CHOP expression was examined in mock-infected; PRRSV-infected; and mCherry-, Nsp3-mCherry-, or Nsp5-mCherry-expressing Marc-145 cells using immunoblotting. The intensities of CHOP bands were normalized to GAPDH. The splicing of the Xbp1 mRNA was examined using PCR; Xbp1u is the unspliced mRNA, and Xbp1s is the spliced mRNA

PRRSV NSP3 and NSP5 are predicted to be transmembrane proteins, and we studied which organelle membrane is the attachment site of these two integral transmembrane proteins. As shown in Fig. 5a and b, PRRSV NSP3-mCherry and NSP5-mCherry only colocalized with calnexin but not with MitoTracker Green. Thus, PRRSV NSP3 and NSP5 are integral transmembrane proteins of the ER but not the mitochondria. The activation of autophagy is always affected by ER stress, which is due to protein processing dysfunctions; the C/EBP homologous protein (CHOP) is overexpressed [38], and the Xbp1 mRNA is spliced by phosphorylated IRE1. PRRSV triggers the activation of the IRE1α and PERK branches of the UPR, suggesting that CHOP overexpression is activated by the PERK pathway and that the autophagosomes induced by PRRSV NSP3 and NSP5 are derived from the ER; therefore, we examined CHOP expression using immunoblotting, and we determined the splicing of the Xbp1 mRNA using PCR. As shown in Fig. 5c, compared to the expression of CHOP in mock-infected cells, this protein was noticeably upregulated in PRRSV-infected cells; however, the expression of CHOP in Nsp3-mCherry- and Nsp5-mCherry-transfected cells was not significantly changed. Thus, the involvement of PRRSV NSP3 and NSP5 in autophagy was not regulated by an ER stress-dependent pathway.

### The cytoplasmic domain is required for PRRSV NSP3-induced autophagy

**Fig. 6:**
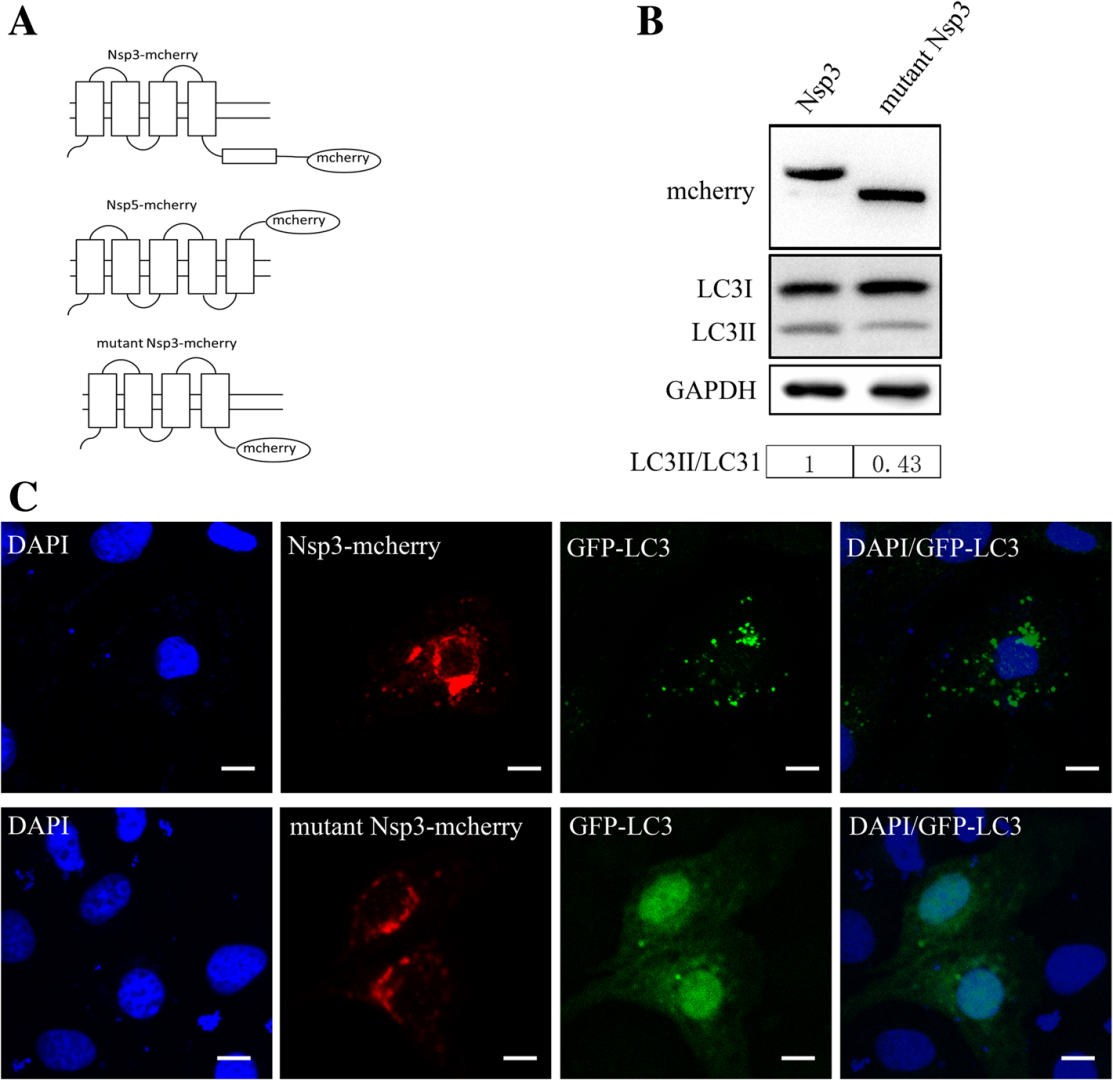
The NSP3 hydrophilic domain is required to activate autophagy. **a** Schematic of the PRRSV NSP3, NSP5 and mutant NSP3 protein structures. **b** mCherry was detected by immunoblotting in Nsp3- and mutant Nsp3-expressing cells **c** Marc-145 cells were co-transfected with plasmids expressing wildtype/mutant PRRSV Nsp3 and GFP-LC3 for 24 h. Fixed cells were detected using fluorescence microscopy. GFP-LC3 puncta represent mature autophagosomes; nuclei were stained with DAPI (blue). Scale bars: 10 μm

PRRSV NSP3 and NSP5 are predicted transmembrane proteins. As shown in Fig. 6a, NSP3 consists of 4 hydrophobic transmembrane domains and a hydrophilic cytoplasmic domain and NSP5 consists of 5 hydrophobic transmembrane domains. A deletion mutant in which NSP3 consisted of only the hydrophobic domains was constructed to detect whether the hydrophilic cytoplasmic domain of NSP3, which is out of range of the membrane, affects the formation of autophagosomes. As illustrated in Fig. 6b, the NSP3 mutant was successfully constructed, and the truncated protein exhibited a lower molecular weight than the normal NSP3 protein. Furthermore, as shown in Fig. 6c, along with the normal NSP3 protein, the mutant NSP3 protein was localized on the ER but did not induce the formation of autophagosomes. These results reveal that the hydrophilic cytoplasmic domain of NSP3 plays an important role in inducing autophagy.

## Discussion

Knowledge about virus-induced autophagy has gradually increased [37]. However, the relationship between each PRRSV protein and autophagy has not been elucidated. As shown in the present study, the induction of autophagy by PRRSV NSP3 and NSP5 contributed to the formation of autophagosomes derived from the ER, and the mature autophagosomes were not degraded by fusion with lysosomes. PRRSV NSP3 and NSP5 are ER transmembrane proteins, but these multiple processes of autophagy were not induced by ER stress. Moreover, a deletion mutant of NSP3 revealed that the transmembrane domains are crucial for inducing the formation of autophagosomes. Our findings offer new explanations for the activation of autophagy by PRRSV NSPs.

Autophagy plays an important role in both the innate and adaptive immune responses, both of which eliminate intracellular microbes [39]. However, many pathogens that invade cells induce the formation of double-membrane autophagosomes to facilitate their own replication [40]. Almost all viruses induce the formation of autophagosomes, and autophagosomes provide the site for viral replication and assembly [41]. Additionally, individual nonstructural proteins or structural proteins may perform viral functions, such as inducing apoptosis or necrosis. In the present study, PRRSV CH-1a induced the formation of autophagosomes in the infected cells; these autophagosomes are purported to be the site of viral replication.

Viral nonstructural proteins play important roles in the replication of the viral RNA and in the synthesis of viral particles. In virus-infected cells, the virus interferes with and destroys the normal metabolism of the host cell, causing cell death or apoptosis [42]. In many studies, viral nonstructural proteins have been shown to play important roles in virus-induced autophagy. In HCV-infected cells, NS3/4A blocks the RIG-I signaling pathway in the early stages of autophagy and modulates the binding of mitochondria-associated immunity-associated GTPase family M (IRGM) to promote autophagy [43]. Similarly, JEV NS3 targets IRGM to modulate autophagy [44], and CBV2 protein 2B, whose C-terminal sequence plays a decisive role in autophagy, rearranges the cell membrane [45]. In our study, GFP-LC3 puncta accumulated in Nsp3-mCherry-, Nsp5-mCherry- and Nsp9-mCherry-transfected cells and p62 expression was increased, indicating that NSP3, NSP5 and NSP9 induced the formation of autophagosomes. Furthermore, we detected an increase in the ratio of LC3II to LC3I in cells transfected with Nsp3 and Nsp5, along with an increased expression of p62, confirming that NSP3 and NSP5 induce autophagy.

Generally, the process of autophagy includes two steps: the formation of autophagosomes and the degradation of autophagosomes. In the early stage of autophagy, ATG5 interacts with ATG12 and ATG16 to form the ATG12-ATG5-ATG16L1 complex; this complex is localized in phagophores, which are the precursors of autophagosomes. In addition, the majority of autophagic degradation occurs when autophagosomes envelope cellular substances, which are delivered to the lysosomes for degradation of the contents. In the present study, a marker of phagosomes, which are the precursor of autophagosomes, namely, ATG5, was colocalized with calnexin, an ER marker, but not with MitoTracker, suggesting that the autophagosomes induced by NSP3 and NSP5 were derived from the ER but not from the mitochondria.

Moreover, autophagy is a complex process. After PRRSV infects cells, it incompletely induces autophagy and inhibits the fusion of autophagosomes with lysosomes. This inhibition leads to the accumulation of autophagosomes and enhances PRRSV replication. In our study, LC3 was not colocalized with LAMP1 in Nsp3- and Nsp5-transfected cells, suggesting that the mature autophagosomes induced by NSP3 and NSP5 were not fused with lysosomes. Our findings provide evidence that the membranes of autophagosomes induced by NSP3 and NSP5 were derived from the ER and inhibited autophagosome fusion with lysosomes.

NSP3 and NSP5 are two transmembrane proteins that appear to play a role in membrane rearrangement. PRRSV NSP3 and NSP5 were localized to the ER, but not the mitochondria, in the present study, suggesting that the two NSPs are transmembrane ER proteins but not transmembrane mitochondrial proteins. In a recent study, ER stress was activated in PRRSV-infected cells. Additionally, researchers previously believed that autophagy activation is accompanied by the activation of the ER stress pathway. The early ER stress response involves the splicing of the Xbp1 mRNA and the expression of CHOP. In the present study, we confirmed that the splicing of the Xbp1 mRNA was not noticeably altered and CHOP expression exhibited a slight increase, indicating that autophagy was not activated by ER stress in PRRSV Nsp3- and Nsp5-transfected cells. Because PRRSV NSP3 contains transmembrane domains and a hydrophilic cytoplasmic domain, we deleted the cytoplasmic domain and revealed that the activation of autophagy requires complete NSP3 protein.

## Conclusions

In conclusion, PRRSV NSP3 and NSP5, which are ER transmembrane proteins, induce the formation of autophagosomes. Although these autophagosomes are derived from the ER and are not degraded by lysosomes, the activation of autophagy by the two NSPs does not involve ER stress. The hydrophilic cytoplasmic domain of PRRSV NSP3 plays a key role in the activation of autophagy. Overall, our findings provide new insights into the connection between autophagy processes and PRRSV NSPs.

## Additional files

Additional file 1: **Table S1.** Table Primers used for PCR. (DOCX 16 kb)

Additional file 2: **Figure S1.** The expression of each PRRSV NSP in Marc-145 cells after transfection using Lipofectamine 3000. Scale bars: 200 μm. (TIF 27251 kb)

## Abbreviations

ER: Endoplasmic reticulum
PRRSV: Porcine reproductive and respiratory syndrome virus
