# Supplementary material for: Involvement of PRRSV NSP3 and NSP5 in the autophagy process
Source: Virol J. 2019 Jan 28;16:13. doi: 10.1186/s12985-019-1116-x (PMC6350329; doi:10.1186/s12985-019-1116-x)
Supplement: Supplementary file 1 — Table S1. Table Primers used for PCR. (DOCX 16 kb) [file 12985_2019_1116_MOESM1_ESM.docx]

| Primers | Primer sequence 5’→ 3’ |
| --- | --- |
| nsp1-F | CCCTCGAGATGTCTGGGATACTTGATCG |
| nsp1-R | CGGGATCCCGCATAGCACACTCAAAAGGGC |
| nsp2-F | CCCTCGAGATGGCTGGAAAGAGAGCAAGGAA |
| nsp2-R | CGGGATCCCGTCCTCCCGAAGGCTTGGAAA |
| nsp3-F | CCCTCGAGATGGGCCCACACCTCATTGC |
| nsp3-R | CGGGATCCCGCTCAAGGAGGGACCCGAGCTGA |
| nsp4-F | CCCTCGAGATGGGCGCTTTCAGAACTCGAAA |
| nsp4-R | CGGGATCCCGTTCCAGTTCGGGTTTGGCAG |
| nsp5-F | CCCTCGAGATGGGAGGCCTTTCCACCGTCCAAC |
| nsp5-R | CGGGATCCCGCTCGGCAAAGTATCGCAAGAAGAAA |
| nsp7-F | CCCTCGAGATGTCGCTGACTGGTGCCCTCGC |
| nsp7-R | CGGGATCCCGCTCCAGAACTTTCGGTGGGA |
| nsp8-F | CCCTCGAGATGGCCGCCAAGCTTTCCGTGGA |
| nsp8-R | CGGGATCCCGCACTGCTCCTTAGTCAGGCC |
| nsp9-F | CCCTCGAGATGGTTTAAACTGCTAGCCGCCA |
| nsp9-R | CGGGATCCCGCTCATGATTGGACCTGAGTT |
| nsp10-F | CCCTCGAGATGGGGAAGAAGTCCAGAATGTG |
| nsp10-R | CGGGATCCCGGCGCTATTTGCGCAGATCTG |
| nsp11-F | CCCTCGAGATGGGGTCGAGCTCCCCGCTCCC |
| nsp11-R | CGGGATCCCGTTCAAGTTGAAAATAGGCCG |
| nsp12-F | CCCTCGAGATGGGCCGCCATTTCACCTGGTA |
| nsp12-R | CGGGATCCCGATTCAGGCCTAAAGTTGGTT |
| nsp3-σR | CGGGATCCCGAACTAATAACACCACGGCCAAGATT |
| xbp1-F | TTACGAGAGAAAACTCACGGCC |
| xbp1-R | GGGTCCAAGTTGTCCAGAATGC |
|  |  |
