# Supplementary figures and images for: Involvement of PRRSV NSP3 and NSP5 in the autophagy process
Source: Virol J. 2019 Jan 28;16:13. doi: 10.1186/s12985-019-1116-x (PMC6350329; doi:10.1186/s12985-019-1116-x)

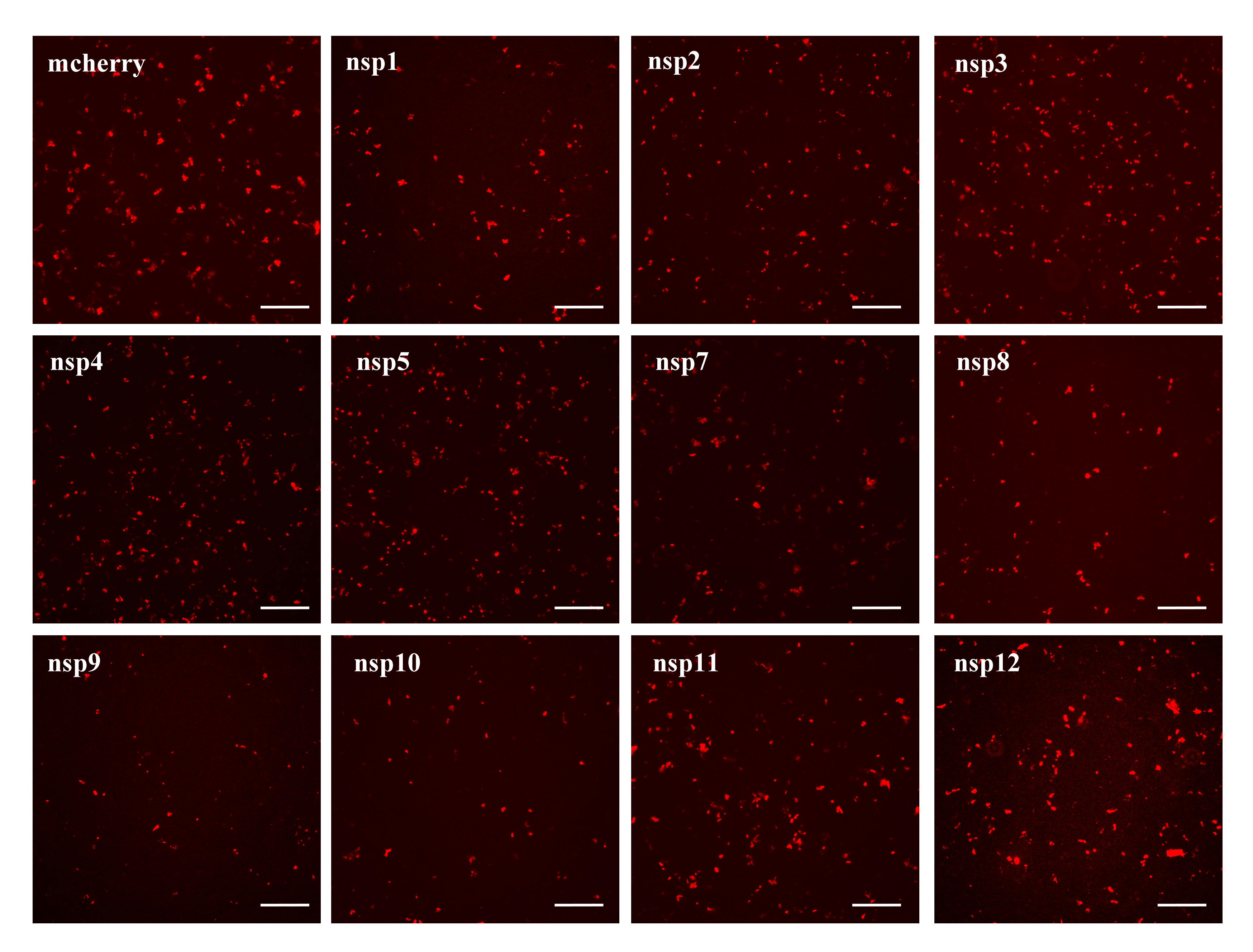

Supplement: Supplementary file 2 — Figure S1. The expression of each PRRSV NSP in Marc-145 cells after transfection using Lipofectamine 3000. Scale bars: 200 μm. (TIF 27251 kb) [file 12985_2019_1116_MOESM2_ESM.tif]
